# Learning more from exploratory trials of complex interventions: exploiting the complexity of complex interventions to empirically optimise the content and delivery of intervention packages for evaluation in a confirmatory trial

**DOI:** 10.1186/1745-6215-16-S2-O80

**Published:** 2015-11-16

**Authors:** Rebecca Walwyn, Steven Gilmour, Amanda Farrin, Allan House

**Affiliations:** 1 University of Leeds, Leeds, UK; 2 University of Southampton, Southampton, UK

## 

MRC guidance is often cited when defining what makes an intervention complex. Here, they are described as ‘interventions that contain several interacting components’. This recognises their multi-component nature but then regards them as *packages* of components. As a result, complex interventions are usually represented by a single treatment variable in the analysis, just as a drug would be. We argue that exploiting the complexity of a complex intervention in a randomised trial could be done by allowing an intervention package to be represented by more than one treatment variable, allowing these to interact. We take the four aspects of intervention complexity highlighted by the MRC; namely (i) number of interacting components, (ii) need to characterise delivery, (iii) degree of tailoring, and (iv) potential levels at which they might work; and map these onto experimental designs, some long-established, with others more recently proposed. Using psychological interventions as an example, we will take each of these four aspects in turn to outline what we believe makes a complex intervention complex from a statistical perspective. It follows that the aim of an exploratory trial should be to understand the fixed and random components of a complex healthcare intervention, for a defined problem or system of problems, which are causally and incidentally important, to what extent, in what combination and circumstances, why and for whom. We will suggest that new experimental designs are needed that simultaneously handle all four of the complexities outlined here to address this aim, discussing how these might be developed.

